# Protocol for the "Michigan Awareness Control Study": A prospective, randomized, controlled trial comparing electronic alerts based on bispectral index monitoring or minimum alveolar concentration for the prevention of intraoperative awareness

**DOI:** 10.1186/1471-2253-9-7

**Published:** 2009-11-05

**Authors:** George A Mashour, Kevin K Tremper, Michael S Avidan

**Affiliations:** 1 University of Michigan Medical School, 1H247 University Hospital, SPC-5048 1500 East Medical Center Drive Ann Arbor, MI 48109-5048, USA; 2 Washington University School of Medicine Department of Anesthesiology Box 8054 660 S. Euclid St. Louis, MO 63110, USA

## Abstract

**Background:**

The incidence of intraoperative awareness with explicit recall is 1-2/1000 cases in the United States. The Bispectral Index monitor is an electroencephalographic method of assessing anesthetic depth that has been shown in one prospective study to reduce the incidence of awareness in the high-risk population. In the B-Aware trial, the number needed to treat in order to prevent one case of awareness in the high-risk population was 138. Since the number needed to treat and the associated cost of treatment would be much higher in the general population, the efficacy of the Bispectral Index monitor in preventing awareness in all anesthetized patients needs to be clearly established. This is especially true given the findings of the B-Unaware trial, which demonstrated no significant difference between protocols based on the Bispectral Index monitor or minimum alveolar concentration for the reduction of awareness in high risk patients.

**Methods/Design:**

To evaluate efficacy in the general population, we are conducting a prospective, randomized, controlled trial comparing the Bispectral Index monitor to a non-electroencephalographic gauge of anesthetic depth. The total recruitment for the study is targeted for 30,000 patients at both low and high risk for awareness. We have developed a novel algorithm that is capable of real-time analysis of our electronic perioperative information system. In one arm of the study, anesthesia providers will receive an electronic page if the Bispectral Index value is >60. In the other arm of the study, anesthesia providers will receive a page if the age-adjusted minimum alveolar concentration is <0.5. Our minimum alveolar concentration algorithm is sensitive to both inhalational anesthetics and intravenous sedative-hypnotic agents.

**Discussion:**

Awareness during general anesthesia is a persistent problem and the role of the Bispectral Index monitor in its prevention is still unclear. The Michigan Awareness Control Study is the largest prospective trial of awareness prevention ever conducted.

**Trial Registration:**

Clinical Trial NCT00689091

## Background

Intraoperative awareness with explicit recall (AWR) is a complication feared by both patients and clinicians alike. In 2004 the Joint Commission on Accreditation of Hospital Organizations issued a Sentinel Alert in order to promote greater attention to the problem [1]. A multi-center study in the United States by Sebel *et al *[2] estimated an incidence of awareness with explicit recall of approximately 0.13%, which is consistent with European studies [3]. A proportion of patients experiencing AWR may subsequently develop serious psychological sequelae, including post-traumatic stress disorder (PTSD) [4,5]. As such, a reliable and practical method of preventing AWR would be an important clinical advance.

The Bispectral Index (BIS) monitor has been proposed as a method of assessing adequate depth of anesthesia and is based on electroencephalography (EEG) analysis of burst suppression, spectral power of the beta bandwidth, and bispectral coherence [6,7]. The proprietary algorithm of the BIS combines these features and expresses the depth of anesthesia by a dimensionless number ranging from 100 (fully awake) to 0 (isoelectric). The BIS monitor has been validated in a number of studies assessing both anesthetic drug concentrations and level of sedation [7-9].

Ekman *et al *[10] prospectively studied 4945 patients who were to receive general endotracheal anesthesia with neuromuscular blockade. These patients were monitored with the BIS, with values recommended between 40 and 60. The incidence of AWR in this population was 0.04%, compared with 0.18% in a historical control group of 7826 patients from a previous study of awareness in which no cerebral function monitoring was used. The incidence of 0.04% in this cohort represented 2 patients, both of whom had BIS values of 60 for 4 minutes or more.

Myles *et al *[11] completed a prospective, randomized study of BIS-guided anesthetic management of patients at high risk for awareness. Postoperative interviews identified 2 patients (0.16%) who experienced confirmed AWR in the BIS-guided group compared to 11 (0.9%) in the control group. One patient with awareness had an episode during which the BIS had reached approximately 80, whereas the other had a period in which the BIS reached 55-59. Collectively, these studies suggested the potential for the BIS to reduce the incidence of intraoperative awareness. A more recent study by Avidan *et al *[12] in the high risk population suggested that a protocol to increase vigilance, rather than the BIS monitor itself, could possibly account for the reduction in the incidence of awareness. They found no statistically significant difference between a BIS-guided and minimum alveolar concentration (MAC)-guided protocol.

Assuming the BIS can reduce the incidence of AWR, the number needed to treat in order to avoid one case of awareness in the high-risk population was 138, with an associated cost of approximately US$2200 in 2004 [11]. The number needed to treat and its associated cost would thus rise considerably in the general population. Furthermore, if one considers the total number of awareness reports ("possible" plus "confirmed"), there was no difference between the BIS and routine care groups in the B-Aware trial. Therefore, the clear efficacy of BIS monitoring in the general surgical population would need to be established before recommending it as a general practice, especially given the results of the B-Unaware trial. This is the basis for conducting the prospective and randomized Michigan Awareness Control Study (MACS).

## Methods/Design

### Ethics

We have received Institutional Review Board approval from the University of Michigan Medical School.

### Calculating Sample Size

Sample size was based on an incidence of AWR of 1-2 cases/1000, in agreement with the studies of Sebel *et al *[2] and Sandin *et al *[3]. To compare an incidence of intraoperative awareness of approximately 0.15% using conventional management *versus *a 0.04% incidence rate with BIS monitoring (a rate based on Ekman *et al *[10]), we calculate a need for 14,072 per group or a total n = 28,144 with an 80% power. Given the potential for missing data or patient non-compliance, we will target at least 30,000 patients for the current study. Based on our current recruitment rate, we estimate that in approximately 36 months we can realistically achieve this sample size at our institution.

### Assessing the Incidence of Awareness

We are assessing the presence of AWR at 28-30 days. Given the extremely large number of patients to be interviewed, we chose only one interview time point. Interviewers blinded to the study conditions conduct the modified Brice interview [13], which is defined by the following questions:

1. What was the last thing you remember before going to sleep?

2. What is the first thing you remember after waking up?

3. Do you remember anything between going to sleep and waking up?

4. Did you dream during your procedure?

5. What was the worst thing about your operation?

Based on the data obtained from these interviews, any reports suggestive of awareness are investigated and classified by a committee blinded to the study conditions. Awareness reports are classified as:

1. No awareness, or awareness of something with a high probability of occurring in the immediate preoperative or postoperative period.

2. Possible awareness: patient unable to recall any event definitively indicative of awareness.

3. Definite awareness in which events are confirmed or have a high likelihood of occurring in the intraoperative period.

Any patients with possible or definite awareness are followed by the primary anesthesiologist, as well as the Quality Assurance division of our department. Psychiatric care is formally offered by Quality Assurance representatives to all patients with possible or definite awareness. Events are also classified according to the Michigan Awareness Classification Instrument, which has been shown to have excellent inter-observer agreement (**Appendix 1**) [14].

### Comparing the BIS-guided and MAC-guided electronic alerts for the prevention of awareness under general anesthesia

We anticipate approximately 30,000 participants over 36 months of study who satisfy the following criteria:

Inclusion

• >18 years of age

• Competent to give informed consent

• Available for follow-up

Exclusion

• Adhesive allergy

• Surgery involving the forehead

• Psychosis or memory impairment

• History of brain injury

• History of electroencephalographic abnormality (e.g., epilepsy or congenital low-voltage EEG)

BIS monitors that do not have independent displays have been introduced into the monitoring modules of adult operating rooms at the University of Michigan (University Hospital, Cardiovascular Center, East Ann Arbor outpatient surgery center). We obtain informed consent from eligible patients undergoing surgery in these rooms, then apply the BIS Quattro sensor to the left forehead according to manufacturer instructions. Myles *et al *[11] and Avidan *et al *[12] have already conducted prospective studies of patients traditionally considered to be at high risk for awareness. Since the risk factors for AWR are still not completely understood--and since it is important to evaluate the efficacy of the BIS monitor in the general population--we are including all surgical patients receiving general anesthesia, regardless of the perceived risk.

The University of Michigan Health System utilizes the Centricity electronic perioperative information system in all of its operating rooms, as well as the post-anesthesia care unit. Using this system, automated real-time analysis of BIS values or MAC levels is possible, with the output of electronic alphanumeric alerts if certain criteria are not met. The most common preventable cause of AWR is insufficient levels of anesthesia [15]. Although MAC and MAC-awake are well known to vary with age and surgical procedure [16], it can be argued that <0.5 age-adjusted MAC is approaching the level where consciousness may return (i.e., MAC-awake) [17]. Operating rooms are randomized to either (1) electronic alerts in the event of BIS values >60, or (2) electronic alerts for age-adjusted MAC-level of <0.5. In addition to the standard inhalational anesthetics, the "effective MAC" level also incorporates documented propofol infusions, dexmedetomidine infusions, and boluses of propofol, sodium thiopental, etomidate, or ketamine (**Appendix 2**) [18]. Alerts state the BIS value or MAC level, followed by "Potentially insufficient anesthesia- please check vaporizers and intravenous lines." The clinicians electronically signed into the case also have the option of suspending the alerting system for a period of 30 minutes after at least 2 pages regarding potentially insufficient anesthesia have been generated. In order to do this, they must electronically enter a reason for suspending pages (such as stimulus-appropriate anesthetic, emergence, etc).

In the BIS-targeted rooms, BIS values appear on the main monitoring screen and are automatically recorded. In the MAC alert-targeted rooms, BIS values will neither appear on the monitor nor be immediately accessible. Other aspects of anesthetic care (e.g., choice of anesthetic agents) are not standardized for this study. Consistent with other studies of awareness and BIS monitoring, the use of benzodiazepines is not standardized.

After data have been gathered on 30,000 patients, the total events of AWR during general anesthesia will be calculated based on the data acquisition described above. Subsequently, the individual cases will be appropriately categorized based on whether they were in the BIS or MAC group, allowing statistical comparison of the incidence of awareness (definite awareness, possible awareness, aggregate) in the two randomized groups. An interim analysis by an independent data monitoring group will be performed after 20,000 patients have been recruited. Asymmetric stopping boundaries will be designed to allow early termination of the trial if the use of BIS-guided alerts is found to reduce AWR or if there is a low probability that the trial can demonstrate a lower AWR rate in the BIS-alert group than in the MAC-alert group (futility stopping rule).

### Secondary Outcomes

Other studies will be conducted based on the data gathered at the University of Michigan, including:

• Comparison of Quality Assurance and prospective assessments of awareness incidence using data from the University of Michigan.

• Incidence of post-traumatic stress disorder (PTSD) in patients with definite or possible AWR.

• Predictors of PTSD based on the classification of the awareness event.

• Incidence and type of dreams during anesthesia in conjunction with MAC or BIS values.

• The relationship of cumulative time with BIS <45 (as well as other BIS thresholds), anesthetic doses, and mortality.

• Relationship between BIS values and hemodynamic parameters.

• Analysis of interrupted monitoring during the use of the BIS.

• Reasons for the purposeful administration of light anesthesia.

• Efficacy of MAC-based alerts in cases with exclusively inhalational anesthesia compared with those including intravenous infusions.

• BIS values and anesthetic dosing of chronic pain patients.

• Changes in overall use of anesthetics before and after the introduction of alerts.

• Overall use of anesthetics comparing the BIS to MAC alerts.

• Postanesthesia care unit pain scores, neurologic exam, and discharge time in relationship to BIS values.

• Incidence of post-operative nausea and vomiting in relationship to BIS values.

## Discussion

Awareness during general anesthesia continues to be an important challenge for the anesthesiologist and a source of major distress for the patient. The BIS monitor is the only processed EEG monitor that has been shown to reduce awareness in a prospective study, but its successful use for the prevention of awareness in the general surgical population has not yet been investigated. MACS is the first prospective, randomized, controlled trial of the BIS monitor and awareness that includes all awareness risk levels and incorporates intravenous agents in a MAC-based protocol. It will also prospectively test other novel methods for the prevention or study of awareness, such as the Michigan Awareness Classification Instrument and an electronic algorithm based on both MAC and intravenous agents.

In conjunction with the ongoing BAG-RECALL study, as well as the prior B-Unaware study, we will conduct pre-specified meta-analyses of approximately 38,000 patients prospectively studied at 4 institutions. This will be especially important for the assessment of relatively rare outcomes such as mortality or PTSD. Furthermore, since MACS will also include the high-risk population, we will be able to select high-risk patients who received only inhalational anesthesia in order to compare the 0.7 age-adjusted MAC threshold (BAG-RECALL) and the 0.5 age-adjusted MAC threshold (MACS). This will be an important study in assessing critical anesthetic levels for risk of awareness. Collectively, the analysis of data from both MACS and BAG-RECALL has the potential for significantly advancing the field of awareness research.

## Abbreviations

AWR: Awareness with Explicit Recall; BIS: Bispectral Index; EEG: Electroencephalography; MAC: Minimum Alveolar Concentration; MACS: Michigan Awareness Control Study; PTSD: Post-traumatic Stress Disorder.

## Competing interests

The authors declare that they have no competing interests.

## Authors' contributions

GAM is the principal investigator of the study and was the primary author of the manuscript. KKT and MSA were involved in study design and made contributions to the manuscript. All authors have read and approved the final version.

## Appendix 1: Michigan Awareness Classification Instrument

**Class 0**: No awareness

**Class 1**: Isolated auditory perceptions

**Class 2**: Tactile perceptions (e.g., *surgical manipulation or endotracheal tube*)

**Class 3**: Pain

**Class 4**: Paralysis (e.g., *feeling one cannot move, speak, or breathe*)

**Class 5**: Paralysis AND pain

An additional designation of "**D**" for distress was also included for patient reports of fear, anxiety, suffocation, sense of doom, sense of impending death, etc.

## Appendix 2: Electronic Algorithm for the Detection of Potentially Insufficient Anesthesia

• Conditions for an "active case" are:

1. data capture is possible (i.e., not a paper record)

2. data capture is active (i.e., "patient in room" has been electronically entered and end-tidal [Et] CO_2 _is detected)

3. case has been identified as a general anesthetic

4. "anesthesia induction end" has already been documented

5. request for recovery room bed or transport to an intensive care unit has not been documented

6. surgical dressing completion has not been documented

• The alerting system checks the most recent value (within a specified time period) of:

1. Et Sevoflurane (MAC = 2.0)

2. Et Isoflurane (MAC = 1.2)

3. Et Desflurane (MAC = 6)

4. Et Nitrous Oxide (MAC = 105)

and compares it to the MAC of each agent. It adds the resulting MAC values together for "current total MAC."

• The system then checks for a charted propofol infusion in mcg/kg/min and divides by 150, assuming that 150 mcg/kg/min is "1.0 MAC" for propofol. The analogous concept of MAC for propofol is "Cp50"- the plasma or blood concentrations at which 50% of patients do not move in response to a noxious stimulus. Since we do not have the technology at our institution to calculate Cp50 or Cp50-awake, we have chosen the above propofol dose as an initial value based on clinical experience. The resultant MAC equivalent is added to current total MAC. (For further discussion, see [18].)

• The system next checks for a dexmedetomidine infusion with a rate of 0.2 mcg/kg/hour or greater. If present, it multiplies the current total inhalational MAC by 2, as dexmedetomidine can reduce MAC by 50% [19].

• At this point, the "current total MAC" is defined as: Et Sevo/2 + Et Iso/1.2 + Et Des/6 + Et Nitrous/105 + propofol rate (in mcg/kg/min)/150. If dexmedetomidine is ≥ 0.2 mcg/kg/hour, inhalational MAC is multiplied by 2.

• If this total MAC is below a set threshold, the system assesses whether a bolus of propofol, midazolam, etomidate, or thiopental has been documented in the preceding 10 minutes.

• The system then triggers an alert if total age-adjusted MAC is below the assigned threshold AND no bolus has been documented in the preceding 10 minutes. Age adjustment for MAC is only performed for volatile agents and is based on calculations derived from prior literature [16,20].

• If implemented, the clinician electronically signed into the case receives an alphanumeric page stating "Potentially insufficient anesthesia, please check vaporizers and intravenous lines."

## Pre-publication history

The pre-publication history for this paper can be accessed here:


